# Observational Study on Antimicrobial Treatment Frequencies and Culling Rates in a Cohort of Dairy Herds in Lodi Province (Italy)

**DOI:** 10.3390/ani16081200

**Published:** 2026-04-15

**Authors:** Viviana Fusi, Emanuele Invernizzi, Valerio Sora, Francesca Zaghen, Flavio Sommariva, Alfonso Zecconi

**Affiliations:** 1 Post-Graduate School in Milk and Milk Products Hygiene and Technology, University of Milan, Via Pascal 36, 20133 Milan, Italy; viviana.fusi@unimi.it; 2 ATS Città Metropolitana di Milano, Distretto Veterinario Basso Lodigiano, Via Trieste 76, 26845 Codogno, Italy; einvernizzi@ats-milano.it; 3 Department of Biomedical, Surgical and Dental Sciences, School of Medicine, University of Milano, Via Pascal 36, 20133 Milan, Italy; valerio.sora@unimi.it (V.S.); francesca.zaghen@unimi.it (F.Z.); 4 Associazione Regionale Allevatori Lombardia, Via Kennedy 30, 26013 Crema, Italy; f.sommariva@aral.lom.it

**Keywords:** antimicrobial treatment, culling rate, dairy herds, defined daily dose, sustainability, one health

## Abstract

In recent years, there have been important regulatory and management changes in livestock farming in Italy and other European countries. The goal of these changes has been to decrease the use of antimicrobials (antimicrobial stewardship). The objective of this observational study was to explore the potential relationship between the decrease in the use of antimicrobials and the rise in the culling of dairy cattle on 210 farms in the Lodi province. Records of antimicrobials used in dairy herds (measured as the daily defined dose animal for Italy—DDDAit) and culling rates were retrieved from the Italian Ministry of Health official databases. These were statistically analyzed to assess the presence of an association between changes in the DDDAit and changes in the culling rates in 2023 and 2024. The results indicated that the DDDAit exhibited minimal fluctuations over the two-year period (3.61% vs. 2.45%). However, there was a notable shift observed in the culling rates, particularly among smaller herds (24.51 vs. 39.27%). The statistical analysis showed a significant correlation between herd size and antimicrobial treatment, with a higher frequency in larger herds. The results of this study indicate that reducing antimicrobial treatments alone may not necessarily result in better animal welfare or herd sustainability, particularly when it is associated with an increase in the culling rate. It also suggests that antimicrobial treatment rates and culling rates derived from a mandatory recording system provide a simple means of verifying the level of herd health management and of assessing the antimicrobial stewardship approach efficacy.

## Simple Summary

In recent years, there has been a growing awareness of the importance of using antibiotics responsibly. This has led to some regulatory and management changes in livestock farming in European countries, as well as the introduction of economic incentives to reduce antimicrobial usage. The decline in antimicrobial usage should be linked to an increase in herd health management. This would be expected to result in a reduction in culling rates because of the decrease in disease incidence, when other factors are unchanged. This observational study, which ran over two years (2023–2024), set out to investigate the presence of this association. This was made possible by the availability of data from the mandatory recording system applied in Italy since 2022. A significant increase in the culling rate not paired with a significant decrease in antimicrobial treatments was observed in smaller herds (<200 animals) when compared to larger herds. The results of this study suggest that antimicrobial treatment rates and culling rates derived from a mandatory recording system provide a simple means of verifying the level of herd health management, and to assess if the antimicrobial stewardship approach is successful.

## 1. Introduction

In recent years, the growing attention to the prudent use of antibiotics has led to important regulatory and management changes in livestock farming in Italy as well as in other European countries [1,2]. Among these, the introduction of the Electronic Treatment Register in 2022 represented a turning point in the traceability and transparency of veterinary treatments carried out in food-producing animals. This tool allows for more rigorous monitoring of treatments at the herd level. Indeed, any veterinary prescriptions should be recorded within 24 h, and this should indicate at least the disease requiring the treatment, the animal involved and the start and end of the treatment protocol [3]. Moreover, the current Common Agricultural Policy (CAP) in Europe is strictly related to a reduction in antimicrobial use as a requirement for financial support [4], thus adding an economic incentive to the reduction in antimicrobial usage.

The number of antimicrobial treatments, within a prudent and rational usage, represents an indicator of the health and welfare of the herd, and it is related to the quality and efficiency of the herd management [5,6], but it does not assess the quantity of antimicrobials applied. The assessment of antimicrobial usage in livestock evolved over time, as a response to the need to evaluate the exact quantity of antimicrobials applied in food-producing animals at local, national and European levels. Initially, this assessment was based on the amount of products sold in the different countries, published in terms of milligrams of active ingredient sold per population correction unit (mg/PCU) [7]. This measure was strongly biased by the availability of these data, and by the level of sales outside a specific country. This led to the development of a more precise assessment based on the defined daily dose for animals [8]. Indeed, in 2016, the European Medicine Agency (EMA) introduced the DDDvet/DCDvet system. The DDDvet represents the defined daily dose per kilogram of live weight in a specific animal species, calculated on an annual basis. Similarly, the DCDvet indicates the estimated average dose per kilogram of animal for the entire treatment cycle, providing a measure of the total number of treatments administered over the course of a year. Both indicators allow for data standardization, making it possible to compare different livestock farming practices and veterinary health systems at the European level.

However, these metrics are based on average dosages adopted at the European level, and they are incomplete for some categories (e.g., long-acting macrolides, intramammary). Furthermore, they do not accurately reflect the dosages prescribed for medicines authorized in a specific country, such as in Italy. For this reason, the Italian Ministry of Health has developed its own standard: the DDDAit, or the daily dose defined for each animal species according to the characteristics of the drugs authorized in Italy. Each veterinary medicine has its own DDDAit, based on the dosage reported in the Summary of Product Characteristics (SPC) [9].

Among other indicators that describe the balance and efficiency of herd management, the culling rate plays an important role, as it reflects the farm’s ability to properly manage its livestock over time. The culling rate can vary greatly depending on the type of farm, the breed, the production strategies, and, above all, the level of management [10,11,12]. The term “culling” refers to the voluntary removal of an animal from production, usually for reasons related to an insufficient production performance; health, reproductive, or behavioral problems; or simply the animal reaching its physiological age limit. An excessively high culling rate may indicate animal welfare problems or an excessive production pressure; conversely, an excessively low rate may suggest scarce availability of replacement. This latter problem may be due to ineffective reproduction or health management, leading to the prolonged retention of animals that are no longer profitable, with negative effects on the overall productivity and profitability [10,12,13].

The culling rate also reflects animal welfare: animals that are removed earlier due to lameness, chronic mastitis, or metabolic or reproductive problems indicate that there are critical issues in management, nutrition, the housing environment, or health prophylaxis. From this perspective, an increase in culling may be an indication of chronic stress or subclinical diseases that are not being properly managed [11,14].

Reducing the culling rate caused by diseases is therefore a priority from an ethical point of view, as it improves the quality of life of the animals. It also has a positive effect from an economic point of view, as every animal that leaves production early represents a loss of investment and future productivity [15,16]. In the context of sustainability, the culling rate therefore becomes an indicator of overall efficiency. Indeed, it is the result of a balance between the planned and physiological exit of animals and the introduction of young animals capable of ensuring production continuity, which is a sign of conscious, long-term management.

In this context, the most advantageous condition would be a reduction in the DDDAit and the maintenance of a “physiological” culling rate. Unfortunately, to the best of our knowledge, there is a lack of knowledge on the association between changes in scientifically measured antimicrobial usage (e.g., DDD) and changes in the culling rates in Italy, as well in most of the countries.

The availability of mandatory national electronic registration systems for both antimicrobial usage and culling rates allowed us to investigate the possible association between the use of antimicrobials and the culling rates of dairy cattle on farms in the cohort of dairy herds of the Lodi province in Italy. Therefore, this observational cross-sectional retrospective study was based on an objective and standardized assessment of antimicrobial treatment protocols and doses, and on official data on the number and type of animals that are culled. The aims of the study were to describe the changes in antimicrobial treatment rates and in the DDAit mean values in a relative homogenous population of dairy herds during a two-year period. Moreover, the potential presence of correlations with changes in the culling rates was explored.

## 2. Materials and Methods

### 2.1. Herd Selection

All the data analyzed in this study were collected for the period 1 January 2023 to 31 December 2024. Herds were selected based on their inclusion in the Integrated Veterinary Information Service (Vetinfo) database (247 herds), which represents all the dairy herds officially registered in the Lodi province. Among these herds, 21 were excluded because they started or terminated their activity in one of the two years considered (2023–2024); thus, the data would be incomplete. Twelve other herds were excluded because they did not record any treatments in either of the two years, and four herds were excluded because the Classyfarm database reported an underestimation of the treatment assessment. Therefore, the final number of herds considered was 210.

### 2.2. Data Retrieval

The herd register reports for the two-year period were downloaded from the Vetinfo portal, while the electronic treatment records were downloaded from Classyfarm, an integrated surveillance system of Italian livestock farms [17]. The Vetinfo register reports, among other information, the total number of animals in each herd, the number of animals >23 months of age, and the date and reason for their exit (cull) from the herd. The Classyfarm report includes all the treatments applied in the herd, the reasons for the treatments, pharmaceutical products, the start and end of the treatment, and the specific animals treated. For the treatment calculation, each injection/inoculation was counted separately rather than entire treatment protocols. These data were used to calculate the DDDAit as described below.

### 2.3. Antimicrobial Treatment Rates

The antimicrobial treatment rates (AMTR%) were calculated by dividing the number of antimicrobial treatments by the mean number of cows in the herd. The rates were also calculated for the major reasons for treatment: intramammary (including dry-cow therapy), respiratory and enteric diseases.

### 2.4. Culling Rate

The number of cattle exits was extracted from the herd register. The culling rate was calculated according to Fetrow et al. [18], by dividing the number of cows culled during the period considered by the average number of females over 23 months of age that were present during the same period.

### 2.5. Defined Daily Dose

The DDDAit was retrieved by the Electronic Treatment Register, where the DDDAit value for each herd was automatically calculated following procedures developed by the Italian Ministry of Health [9]. Practically, the DDD represents the dose in milligrams of active ingredient used to treat one kilogram of live weight over a period of twenty-four hours. This dose represents the correct dosage, as defined in the summary of product characteristics (SPC). This dose is identified and separated for each active ingredient present in veterinary antimicrobial drugs that can be used in the species included in the monitoring system. In our case, the species monitored were dairy animals, and the SPC to be considered was the antimicrobial treatments for dairy animals. The DDDAit refers to a pharmaceutical product with its SPC available in Italy. The formula applied to calculate the herd DDDAit in the Classyfarm system is the following [9]:

$$\sum_{i=1}^{n}\frac{Active\;principle\;consumed}{{DDDAit}_{i}\;\left(\frac{mg}{kg\;live\;weight\;per\;day}\;\right)\times animals\;\left(n\right)\times standard\;weight}$$

### 2.6. Statistical Analysis

All the data were collected in an Excel™ (Microsoft, Redmond, WA, USA) database, and the statistical analysis was performed using the appropriate procedures of SAS 9.4 (SAS Institute, Cary, NC, USA).

A general linear model (GLM) was applied to assess the variability in the following response variables: the DDDAit, culling rate, antimicrobial treatment rate (number of treatments/animals in the herds), proportion of intramammary treatments, enteric and respiratory disease treatments, and total antimicrobial treatments. The factors included in the model were the herd size, the year and the interaction between herd size and year.

To assess the relationships among the different parameters considered, a principal component analysis (PCA) was applied (proc FACTOR). A principal component analysis is a multivariate technique for examining relationships among several quantitative variables [19]. In this study, these variables are represented by the herd size, DDDAit, culling rate, antimicrobial treatment rate, proportion of intramammary treatments, enteric and respiratory disease treatment, and total antimicrobial treatments.

## 3. Results

### 3.1. Herd Characteristics

**Figure 1:**
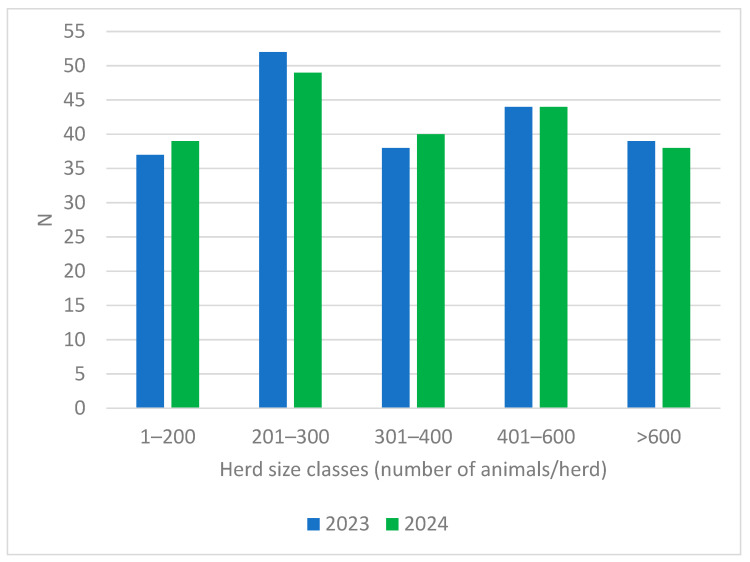
Distribution of the herd size of the 210 dairy herds considered during 2023 and 2024, clustered into five clusters of a similar size.

The study involved 210 dairy herds of the Lodi province in Lombardy (Italy), and their characteristics are reported in Table 1 and in Figure 1. These herds had mainly Italian Holstein cows. The mean and median of the animals in these herds were similar in 2023 and 2024, being statistically not significant. The range between the minimum and maximum widened between 2023 and 2024, but 50% of the herds were in the range of 235–497 animals in both years.

### 3.2. Antimicrobial Treatment Rate

The antimicrobial treatment rates (AMTR%) showed a decrease in 2024 vs. 2023 (Table 2). The mean in 2023 was higher than 100%, as well as the third quartiles in both years. These values were due to recording methods that count each single injection/inoculation and not the whole treatment protocols, which usually include more than one injection/inoculation.

**Figure 2:**
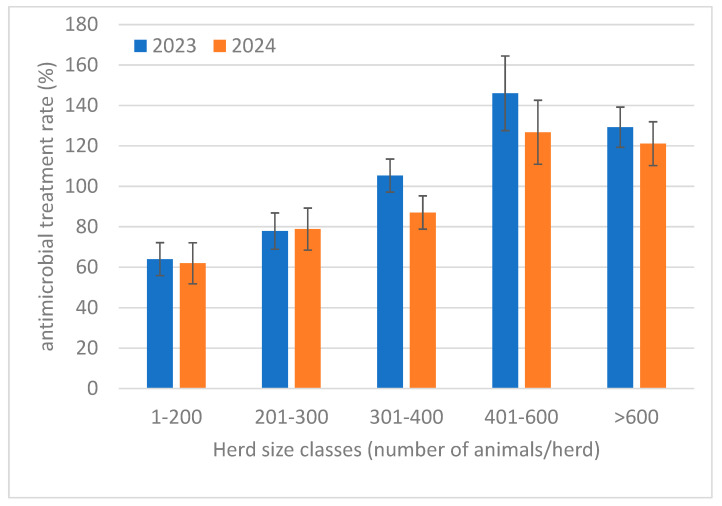
Distribution of antimicrobial treatment rates (mean ± std.err.) for the 210 dairy herds considered during 2023 and 2024 by herd size cluster.

The distribution of the AMTR% by herd size classes (Figure 2) confirmed the decrease in the treatment rates in all classes, but it also showed that the larger herds had significantly higher treatment rates when compared with smaller herds. Indeed, the statistical analysis (Table 3) showed that herds with 400 cows had a higher AMTR% than smaller herds in both 2023 and 2024. However, despite the numerical differences, none of the comparisons between years within each herd size class proved to be significant. The overall cure rate numerically decreased in 2024, but the difference was not statistically significant.

**Figure 3:**
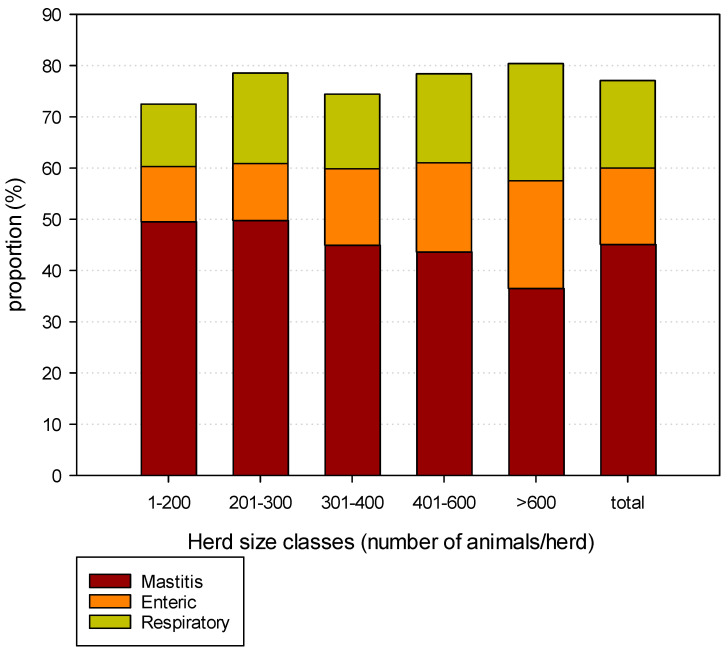
Distribution of proportion of treatments by pathologies for the 210 dairy herds considered during 2023 by herd size classes.

Figure 3 describes the proportion of the major reasons for treatment—intramammary (including dry-cow therapy), respiratory and enteric diseases—in 2023. These three reasons represented nearly 80% of the AMTR% in all the herds considered for both years. Intramammary antimicrobial treatments were the major reason for the AMTR% in all herds, but principally in smaller herds, while the largest proportion of the AMTR% due to enteric and respiratory diseases was observed in larger herds (>600 animals).

Table 4 reports the statistical analysis of the variance in the AMTR% proportions for the three major diseases in the five herd size classes. An overall significant decrease in proportion was observed only for respiratory diseases between 2023 and 2024, and a numerical decrease for enteric diseases. The statistical analysis confirmed that the proportions of treatment for enteric and respiratory diseases were statistically higher in herds with more than 400 cows, while the intramammary treatment proportion was significantly lower in larger herds.

### 3.3. Defined Daily Dose

The change in the treatment rates is only one of the parameters that can be used to assess the changes in the application of the AMTR% in the dairy sector. The calculation of the DDDAit is another parameter, and it is considered a more accurate measure of the amount of antimicrobials delivered to animals [9].

The availability of the DDDAit for the 210 herds considered allows the changes in this parameter to be described over the two years considered. Table 5 reports the statistics for this measure in the two years considered. A small numerical reduction in both the mean and median was observed, but the mean reduction was shown to be non-significant when the GLM model was applied.

**Figure 4:**
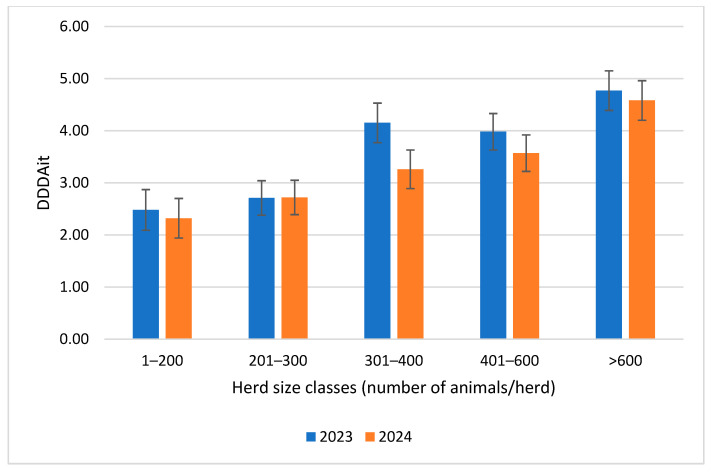
Distribution of the DDDAit (mean ± std.err.) for the 210 dairy herds considered during 2023 and 2024 by herd size classes.

The statistical analysis of changes in the DDDAit for each herd size class over the two years (Figure 4 and Table 6) revealed that the DDDAit values were significantly higher in herds with more than 400 animals in both 2023 and 2024. However, only herds in class 301–400 showed a significant decrease in the DDDAit between 2023 and 2024. A numerical, non-significant reduction was also observed for all other clusters except the 201–300 cluster.

### 3.4. Culling Rates

The culling rate statistical description for the two years is reported in Table 7. The mean value was 27% in 2023 and 30% in 2024 with a relative increase of 10%. The median slightly increased as well as the maximum value.

**Figure 5:**
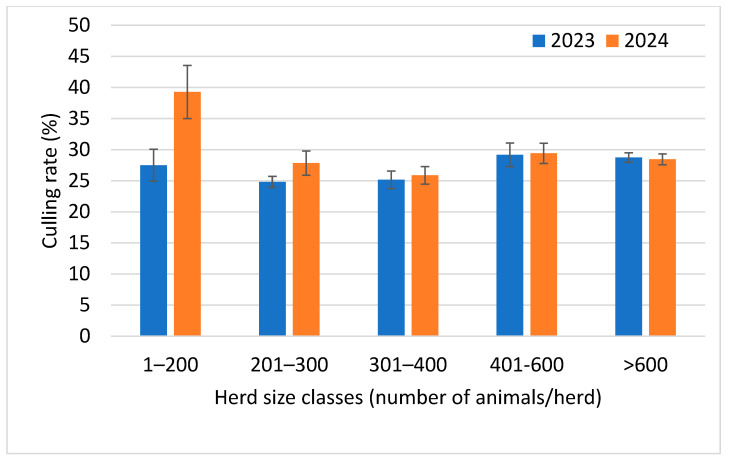
Distribution of culling rate (mean ± std.err.) for the 210 dairy herds considered during 2023 and 2024 by herd size classes.

The analysis of the distribution of culling rates by year and herd size classes (Figure 5) showed a marked increase in the culling rate in herds with <200 animals, growing from about 27% in 2023 to over 40% in 2024. All the other herds showed small increases in the culling rates, with the exception of herds with >600 animals, which showed a very small decrease.

The statistical analysis (GLM) showed that the culling rate in 2024 was significantly higher than in 2023 (*p* < 0.05) (Table 8). This difference was mainly due to the large increase in the culling rate observed in herds with <200 animals in 2024, which was also significantly higher than in all the other herd size classes in the same year.

**Figure 6:**
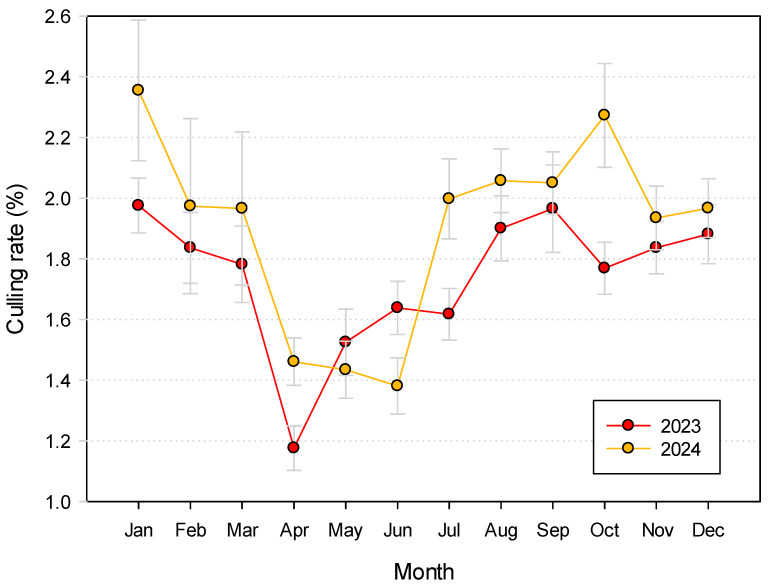
Distribution of culling rate during 2023 and 2024 by month of the year.

Figure 6 reports the culling rates by month for both the years considered. The pattern was similar for both years, with a drop in the culling rates during the April–June trimester, which is generally the period of a higher milk yield. However, the rates were numerically higher during 2024, with very few exceptions (May, June), and with two peaks in January and in October in 2024 that were not observed in 2023.

### 3.5. Principal Component Analysis

To assess the relationships among the different parameters considered, including the herd size, DDDAit, culling rate (CR%), antimicrobial treatment rate (AMTR%), proportion of enteric disease treatments (EDTProp%), proportion of respiratory disease treatments (RDTProp%) and proportion of intramammary treatments (IMTProp%), a principal component analysis (PCA) was applied.

Table 9 shows the correlation among these factors for the year 2023 data. A significant positive correlation was observed between the herd size and the DDDAit, AMTR% rate, and proportion of RDTProp%, and a negative correlation was observed with IMTProp%. The intramammary treatment rate was also negatively correlated with EDTProp% and RDTProp%. The culling rate was not significantly associated with any of the other parameters.

The same analysis performed on the 2024 dataset showed a similar pattern (Table 10), but the correlation between EDTProp% and RDTProp% increased to 1, and the culling rate correlation values were negative with all the other parameters; also, in this case, they were not significant.

The eigenvalues for the 2023 and 2024 datasets are reported, respectively, in Table 11 and Table 12. Seven combinations of factors (herd size, DDDAit, CR%, AMTR%, EDTProp%, RDTProp%, IMTProp%) were identified in 2023 and six in 2024. Moreover, in 2023, the first two factors accounted for 48.5% of the total variance, while the first four factors accounted for 79.1%. In 2024, the first two factors accounted for 58.2% of the variance and the first three factors accounted for 72.5% of the variance.

**Figure 7:**
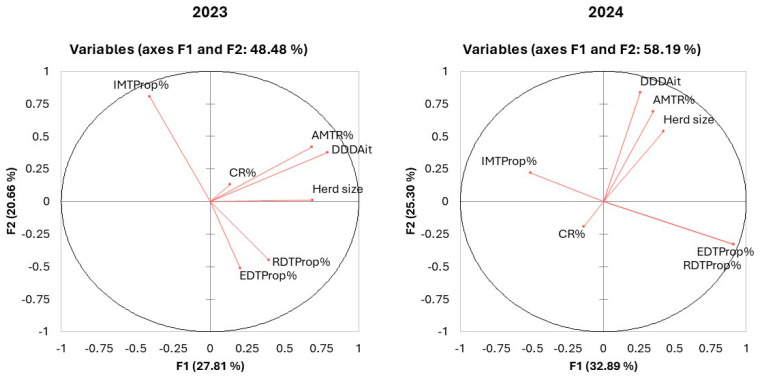
Graphical description of the correlation for the seven parameters included in the F1 and F2 combinations, as analyzed by a principal component analysis. CR%: culling rate, AMTR%: antimicrobial treatment rate, EDTProp%: proportion of enteric disease treatments, RDTProp%: proportion of respiratory disease treatments, IMTProp%: proportion of intramammary treatments. The distance between factors represents their correlation. Close factors are highly correlated, while factors in opposite quadrants are negatively correlated.

The analysis of the results of the principal factor analysis concerning the first two factor combinations for both the 2023 and 2024 datasets are reported in Table 13. The F1 combination in 2023 can be considered a measure of the AMTR% applied in combination with herd size. Indeed, the herd size, DDDAit and AMTR% rate were shown to have the highest values within F1. Furthermore, F2 may be considered a measure of the distribution of treatments based on the disease, with a negative correlation between IMTProp% and the other two treatment categories (EDTProp% and RDTProp%). When the 2024 dataset was considered, we observed that the correlations were different compared to 2023. Indeed, F1 may represent a measure of the proportion of treatments by disease and herd size, while F2 may be considered a measure of the overall treatments and herd size. It should be noticed that, also in this case, there was a negative correlation between IMTProp% and the other two treatment categories. Moreover, in 2024, the culling rate had negative correlation values with the other parameters. Figure 7 describes graphically the previous results and the correlations among the different parameters included in F1 and F2 for the 2023 and 2024 datasets, supporting the previous consideration.

## 4. Discussion

This observational study included all the active herds of an area where dairy production is well established, and the availability of mandatory records for herd characteristics and veterinary treatments supplies a consistent base for epidemiological analyses. This study has some limitations, mainly related to a possible misclassification in the treatment records. The potential availability of other information not included in this study, such as farm management, housing, and disease prevalence, may give more information on the correlation between these latter factors and the treatments and culling rate.

The analysis of the mean size of dairy farms in the Lodi province over the two-year period demonstrated significant stability. The absence of significant differences suggests that, despite any economic, regulatory, or health pressures, the average number of animals raised on farms has not undergone any marked changes in the short term.

Antimicrobial treatment rates numerically decreased in 2024 vs. 2023, with a frequency < 100%. Such high values are due to the method of counting treatments based on the sum of any single doses supplied, and not on the number of treatment protocols. A significant difference in the AMTR% was not observed among the different herd size classes, suggesting the absence of a change in the overall treatment strategy in the different herds. However, a significant difference in the AMTR% was observed for both years considered, between herds with more than 400 animals and smaller ones. Indeed, the AMTR% on average was 68% and 63% higher in large herds compared to smaller ones, respectively, in 2023 and 2024. These differences may be the results of a higher frequency of diseases, a higher attention to their presence or a different attitude to antimicrobial treatments, as suggested by other studies [20,21,22,23]. Unfortunately, it was not feasible to have a consistent and standardized protocol to detect diseases in the dairy herds considered in this study, unless they require treatment, as observed in other studies [21,24,25,26].

The proportion of the AMTR% by disease confirms that mastitis during lactation or at drying off represents the major reason for treatment. The proportion of these treatments was significantly more frequent in smaller herds than in herds with >600 animals. This latter information suggests that management practices in larger herds allow for a reduction in the mastitis prevalence during both lactation and the drying-off period [27,28,29].

The comparison of IMTProp% for the two years showed very similar values, and the absence of statistical differences was confirmed for all the herd size clusters, suggesting that there were no changes in either the importance of the problem or the farmers’ attitude about treating them. The opposite was observed for the proportion of the AMTR% related to RDTProp% which showed a significant decrease in 2024 vs. 2023. Similarly, a decrease in EDTProp% was observed, but the difference was not significant. The absence of changes in the herd characteristics and in the disease frequencies in the two years considered suggests that farmers were probably more focused on calf health management, to decrease the use of antimicrobials at the herd level. For both the diseases and the years considered, the proportions of treatments were significantly higher in herds with >400 animals, with the exception of the proportion of RDTProp% in the 201–300 herd cluster. These figures are the opposite of those observed for IMTProp%, suggesting that calf diseases are more frequent in larger herds.

These results were confirmed by the analysis of the DDDAit, which is a key indicator for monitoring antimicrobial use at the farm level [30,31]. Although there was a slight decrease in the overall average between the two years, this difference was not significant. This could suggest a slight downward trend in antimicrobial use, but it is not yet sufficient to represent a consistent change. The most intriguing data emerged from the substantial variability in the DDDAit observed between herd size clusters. Larger farms (>400 animals) exhibited significantly higher DDDAit values in comparison to smaller farms in both years. This discrepancy may be indicative of different management methodologies, particularly in the context of health management. It may be hypothesized that a significant number of animals and their relative density could increase morbidity and, consequently, the necessity for more frequent treatment. However, it should be noted that many of the risk factors are also present in smaller farms, which, among other things, may have higher animal densities per unit area than larger ones.

Notwithstanding the factors implicated in this reduction, it is expected that a concomitant improvement in other indicators, such as the culling rate, will be observed. However, this improvement has not been observed. Indeed, the mean culling rate increased significantly in 2024 compared to 2023, mainly for the changes observed in smaller herds. Indeed, the culling rate in smaller herds increased by more than 40% in 2024 vs. 2023, while it was nearly unchanged in herds with >300 animals, suggesting a change in farmers’ management choices in smaller herds. This significant increase can be attributed to various structural, managerial, and strategic dynamics. On small farms, cows experiencing reproductive, productive, or health issues may be more likely to be culled, even in the absence of a formal replacement program. This choice may also be attributed to economic constraints: the maintenance of a cow with chronic problems in lactation involves costs that are unsustainable for small farms with lower margins [32,33,34]. The absence of adequately sized facilities dedicated to health management (e.g., infirmary boxes) may further encourage the early culling of diseased animals.

The presence of CAP subsidies related to the DDDAit could be an underlying factor in the decision to cull animals rather than treat them, in order to comply with limits on subsidy allocation. If this relationship is demonstrated, it will be concerning, since decreasing antimicrobial treatments will negatively impact animal welfare. This potential negative link supports the efforts to improve herd health management and decrease the disease frequency and, consequently, antimicrobial treatments [21,35]. Indeed, the contemporary presence of an increase in the CR% in small herds from one year to the next, as well as the lack of significant differences in larger herds, with the significantly higher AMTR% observed in large herds when compared with smaller ones, could indicate greater management and production stability. It can also be hypothesized that large herds have less dependence on CAP subsidies than smaller ones.

However, the choice to cull diseased animals rather than treat them has an impact on animal welfare and the sustainability of the farm that is much higher in small than in large herds [10,36]. Every culled animal represents a lost investment because of the costs incurred during the breeding and production phases (feed, healthcare, fertilization, etc.), which are not recovered, resulting in an economic loss [15,16]. Good economic sustainability therefore requires maximizing the productive life of animals so that the initial costs are recouped and a positive margin is achieved.

The role of herd size and the related health management approach was confirmed by a PCA analysis that showed the correlation among herd size and the different treatment-related parameters. Moreover, a change in the variance explained by the factors related to the proportion of treatments was observed between 2023 and 2024. Indeed, F2 in 2024 explained 38% of the variability, while it was only 20% in 2023. This result suggests that changes in antimicrobial frequency and quantity (DDDAit) were more related to a change in the attitude than to a real and general decrease in the number of treatments. The significant and large reduction in the treatment of respiratory diseases seems to be one of the strongest drivers of these changes.

The present study examined official records pertaining to herd size, antimicrobial usage and culling rates. The absence of a significant decrease in antimicrobial treatment rates and amounts, despite all the efforts of health authorities, veterinarians and herd advisors in addition to the new regulations, raises some concerns. Particularly, the absence of a reduction in antimicrobial usage in large herds and the significant increase in culling rates in smaller herds suggest that a more targeted approach is needed. If a larger dependency on CAP funding for the survival of small herds is confirmed, it could lead to behavior that significantly impairs the welfare of the animals and the overall sustainability of the herd.

## 5. Conclusions

The rational and judicious application of antimicrobial therapy is obligatory for farmers and veterinarians alike, resulting in a reduction in antimicrobial usage. This should result from improved herd health management and should not affect the sustainability of the herd. In a production system where population data recording is mandatory (e.g., treatments, births and deaths), indicators such as the culling rate, antimicrobial treatment rate, and DDDAit considered together may provide a simple means of assessing the level of herd health management.

## Figures and Tables

**Table 1:** Summary statistics of characteristics on the total number of animals in the 210 herds considered in the study.

Year	Minimum	Maximum	1st Quartile	Median	3rd Quartile	Mean	Std.dev.
2023	39	1984	236	339	490	424	324
2024	10	2078	234	333	501	423	327

**Table 2:** Summary statistics of antimicrobial treatment rate parameters in year 2023 and 2024 for the 210 herds considered.

Year	1st Quartile	Median	3rd Quartile	Mean	Std.dev
2023	51.19	86.65	138.17	104.50	81.32
2024	45.76	78.42	130.48	95.15	78.22

**Table 3:** Comparisons of antimicrobial treatment rates during 2023 and 2024 resulting from GLM analysis of variance.

Year	Herd Size					
	1–200	201–300	301–400	401–600	>600	Total
2023	63.99 ^ax1,2^	77.89 ^ax^	105.33 ^ay^	146.01 ^ay^	129.27 ^ay^	104.50 ^a^
2024	61.95 ^ax^	78.87 ^ax^	87.06 ^ax^	126.74 ^ay^	121.12 ^ay^	95.15 ^a^

^1^ Different superscripts (a,b) represent a statistical difference among rows (*p* < 0.05); ^2^ Different superscripts (x,y) represent a statistical difference among columns (*p* < 0.05).

**Table 4:** Comparisons of the proportion of antimicrobial treatment rates by type of disease and herd size classes during 2023 and 2024, evaluated by GLM analysis of variance.

Disease	Year	Herd Size					
		1–200	201–300	301–400	401–600	>600	Total
Enteric	2023	10.78 ^ax1,2^	11.11 ^ax^	14.91 ^ax^	17.38 ^ay^	21.02 ^ay^	15.04 ^a^
	2024	8.13 ^ax^	9.25 ^ax^	11.84 ^ax^	14.70 ^ay^	17.98 ^ay^	12.38 ^a^
Respiratory	2023	12.18 ^ax^	17.65 ^axy^	14.55 ^ax^	17.39 ^ax^	22.86 ^ay^	16.93 ^a^
	2024	8.13 ^ax^	9.25 ^bx^	11.84 ^ay^	14.69 ^ayz^	17.97 ^az^	12.38 ^b^
Mammary	2023	49.54 ^ax^	49.79 ^ax^	44.96 ^ax^	43.64 ^ax^	36.51 ^ay^	44.89 ^a^
	2024	48.34 ^ax^	49.50 ^ax^	43.81 ^ax^	44.66 ^ax^	38.56 ^ay^	44.98 ^a^

^1^ Different superscripts (a,b) represent a statistical difference among rows (*p* < 0.05); ^2^ Different superscripts (x,y,z) represent a statistical difference among columns (*p* < 0.05).

**Table 5:** Summary statistics of the DDDAit parameters in years 2023 and 2024 for the 210 herds considered.

Year	1st Quartile	Median	3rd Quartile	Mean	Std.dev
2023	1.937	2.861	4.560	3.61	2.48
2024	1.702	2.683	3.981	3.29	2.45

**Table 6:** Comparisons of DDDAit based on herd size class during 2023 and 2024, evaluated by GLM analysis of variance.

Year	Herd Size					
	1–200	201–300	301–400	401–600	>600	Total
2023	2.48 ^ax1,2^	2.71 ^ax^	4.15 ^ay^	3.98 ^ay^	4.77 ^ay^	3.61 ^a^
2024	2.32 ^ax^	2.72 ^ax^	3.26 ^bx^	3.57 ^ay^	4.58 ^az^	3.29 ^a^

^1^ Different superscripts (a,b) represent a statistical difference among rows (*p* < 0.05); ^2^ Different superscripts (x,y,z) represent a statistical difference among columns (*p* < 0.05).

**Table 7:** Summary statistics of culling rate parameters in years 2023 and 2024 for the 210 herds considered.

Year	1st Quartile	Median	3rd Quartile	Mean	Std.dev
2023	21.4	25.8	30.0	27.0	10.3
2024	23.1	26.6	30.2	30.0	15.4

**Table 8:** Comparisons of culling rates based on herd size class during 2023 and 2024, evaluated by GLM analysis of variance.

Year	Herd Size					
	1–200	201–300	301–400	401–600	>600	Total
2023	27.51 ^ax1,2^	24.81 ^ax^	25.16 ^ax^	29.18 ^ax^	28.74 ^ax^	27.08 ^a^
2024	39.27 ^bx^	27.84 ^ay^	25.87 ^ay^	29.41 ^ay^	28.45 ^ay^	30.17 ^b^

^1^ Different superscripts (a,b) represent a statistical difference among rows (*p* < 0.05); ^2^ Different superscripts (x,y) represent a statistical difference among columns (*p* < 0.05).

**Table 9:** Correlation matrix for year 2023 data among the seven parameters considered.

Variables	Herd Size	DDDAit	CR% ^1^	AMTR% ^2^	EDTProp% ^3^	RDTProp% ^4^	IMTProp% ^5^
Herd size	1	0.410 *	0.068	0.236 *	0.106	0.191 *	−0.147 *
DDDAit	0.410 *	1	0.046	0.577 *	−0.008	0.127	−0.039
CR%	0.068	0.046	1	0.078	0.029	−0.033	0.010
AMTR%	0.236 *	0.577 *	0.078	1	0.033	0.014	−0.022
EDTProp%	0.106	−0.008	0.029	0.033	1	−0.156 *	−0.390 *
RDTProp%	0.191 *	0.127	−0.033	0.014	−0.156 *	1	−0.410 *
IMTProp%	−0.147 *	−0.039	0.010	−0.022	−0.390 *	−0.410 *	1

^1^ CR%: culling rate; ^2^ AMTR%: antimicrobial treatment rate; ^3^ EDTProp%: proportion of enteric disease treatments; ^4^ RDTProp%: proportion of respiratory disease treatments; ^5^ IMTProp%: proportion of intramammary treatments; * Values are different from 0 with a significance level of alpha = 0.05.

**Table 10:** Correlation matrix for year 2024 data among the seven parameters considered.

Variables	Herd Size	DDDAit	CR% ^1^	AMTR% ^2^	IMTProp% ^3^	EDTProp% ^4^	RDTProp% ^5^
Herd size	1	0.420 *	−0.126	0.243 *	−0.121	0.150 *	0.150 *
DDDAit	0.420 *	1	−0.086	0.556 *	0.017	−0.019	−0.019
CR%	−0.126	−0.086	1	−0.054	−0.035	−0.056	−0.056
AMTR%	0.243 *	0.556 *	−0.054	1	−0.012	0.108	0.108
IMTProp%	−0.121	0.017	−0.035	−0.012	1	−0.338 *	−0.338 *
EDTProp%	0.150 *	−0.019	−0.056	0.108	−0.338 *	1	1.000 *
RDTProp%	0.150 *	−0.019	−0.056	0.108	−0.338 *	1.000 *	1

^1^ CR%: culling rate; ^2^ AMTR%: antimicrobial treatment rate; ^3^ IMTProp%: proportion of intramammary treatments; ^4^ EDTProp%: proportion of enteric disease treatments; ^5^ RDTProp%: proportion of respiratory disease treatments; * Values are different from 0 with a significance level of alpha = 0.05.

**Table 11:** Eigenvalues and proportion of variance explained by the factors’ combination in 2023.

	F1	F2	F3	F4	F5	F6	F7
Eigenvalue	1.947	1.447	1.173	0.972	0.736	0.382	0.343
Variability (%)	27.811	20.665	16.760	13.886	10.521	5.456	4.901
Cumulative%	27.811	48.475	65.235	79.121	89.642	95.099	100.000

**Table 12:** Eigenvalues and proportion of variance explained by the factors’ combination in 2024.

	F1	F2	F3	F4	F5	F6
Eigenvalue	2.302	1.771	0.999	0.841	0.700	0.386
Variability (%)	32.892	25.296	14.271	12.018	10.001	5.521
Cumulative%	32.892	58.188	72.460	84.478	94.479	100.000

**Table 13:** Results of the principal factor analysis and factor-loading correlation for the first two factors for the 2023 and 2024 datasets.

	2023		2024	
	F1	F2	F1	F2
Herd size	0.688	0.008	0.420	0.541
DDDAit	0.790	0.376	0.261	0.835
Culling rate	0.132	0.131	−0.137	−0.192
Antimicrobial treatment rate	0.686	0.420	0.351	0.690
Proportion of enteric disease treatments	0.202	−0.512	0.911	−0.330
Proportion of respiratory disease treatments	0.394	−0.448	0.911	−0.330
Proportion of intramammary treatments	−0.408	0.805	−0.508	0.221

## Data Availability

The data are not publicly available due to privacy regulations concerning treatment and culling records.

## Abbreviations

The following abbreviations are used in this manuscript:

AMTR%	antimicrobial treatment rate
CAP	Common Agricultural Policy EU
DCDvet	estimated average dose per kilogram of animal
DDDAit	daily defined dose for Italy
DDDvet	defined daily dose per kilogram of live weight in a specific animal species
EDTProp%	proportion of enteric disease treatments
EMA	European Medicine Agency
IMTProp%	proportion of intramammary treatments
RDTProp%	proportion of respiratory disease treatments
CR%	Culling rate
